# MiR-429 Regulated by Endothelial Monocyte Activating Polypeptide-II (EMAP-II) Influences Blood-Tumor Barrier Permeability by Inhibiting the Expressions of ZO-1, Occludin and Claudin-5

**DOI:** 10.3389/fnmol.2018.00035

**Published:** 2018-02-07

**Authors:** Liangyu Chen, Yixue Xue, Jian Zheng, Xiaobai Liu, Jing Liu, Jiajia Chen, Zhen Li, Zhuo Xi, Hao Teng, Ping Wang, Libo Liu, Yunhui Liu

**Affiliations:** ^1^Department of Neurosurgery, Shengjing Hospital of China Medical University, Shenyang, China; ^2^Liaoning Clinical Medical Research Center in Nervous System Disease, Shenyang, China; ^3^Key Laboratory of Neuro-oncology in Liaoning Province, Shenyang, China; ^4^Department of Neurobiology, College of Basic Medicine, China Medical University, Shenyang, China; ^5^Key Laboratory of Cell Biology, Ministry of Public Health of China, China Medical University, Shenyang, China; ^6^Key Laboratory of Medical Cell Biology, Ministry of Education of China, China Medical University, Shenyang, China

**Keywords:** blood-tumor barrier, EMAP-II, miR-429, p70S6K, s6K

## Abstract

The blood-tumor barrier (BTB) hinders delivery of chemotherapeutic drugs to tumors in the brain; previous studies have shown that the BTB can be selectively opened by endothelial monocyte activating polypeptide-II (EMAP-II), but the specific mechanism involved remains elusive. In this study, we found that microRNA-429 (miR-429) expression in glioma vascular endothelial cells (GECs) was far lower than in human brain microvascular endothelial cells (ECs). miR-429 had lower expression in GECs and glioma tissues compared to ECs or normal tissues of the brain. Furthermore, miR-429 had lower expression in high grade glioma (HGG) than in low grade glioma (LGG). In *in vitro* BTB models, we also found that EMAP-II significantly increased BTB permeability, decreased expression of ZO-1, occludin and claudin-5 in GECs, in a time- and dose-dependent manner. EMAP-II greatly increased miR-429 expression in GECs of the BTB models *in vitro*. Overexpression of miR-429 in GECs significantly decreased the transepithelial electric resistance (TEER) values in BTB models, and led to enhanced horseradish peroxidase (HRP) flux. Overexpression of miR-429 in GECs significantly decreased the expression of tight junction (TJ)-associated proteins (ZO-1, occludin and claudin-5), and decreased the distribution continuity. Silencing of miR-429 in GECs increased the expression of TJ-associated proteins and the distribution continuity. The dual-luciferase reporter assay revealed that ZO-1 and occludin were target genes of miR-429, and we demonstrated that miR-429 overexpression markedly down-regulated protein expression of p70S6K, as well as its phosphorylation levels. The dual-luciferase reporter assay also showed that p70S6K was a target gene of miR-429; miR-429 overexpression down-regulated expression and phosphorylation levels of p70S6K, and also decreased phosphorylation levels of S6 and increased BTB permeability. Conversely, silencing of miR-429 increased the expression and phosphorylation levels of p70S6K, and increased phosphorylation levels of S6, while decreasing BTB permeability. In conclusion, the results indicated that EMAP-II caused an increase in miR-429 expression that directly targeted TJ-associated proteins, which were negatively regulated; on the other hand, miR-429 down-regulated the expression of TJ-associated proteins by targeting p70S6K, also negatively regulated. As a result, the BTB permeability increased.

## Introduction

The BTB hinders drug delivery to the brain, which makes systematic drug treatment of brain tumors ineffective (Park et al., 2016; Salvador et al., 2016). The BTB is more permeable than the BBB, but it still reduces the delivery of anti-tumor drugs to tumor tissues in the brain (Black and Ningaraj, 2004). The BTB hinders hydrophilic molecules diffusing by paracellular transport into tumor tissues by endothelial TJ composed of integral membrane proteins (claudins, occludin, and junctional adhesion molecules) and intracellular proteins (ZO-1 and cingulin) (Miyoshi and Takai, 2005; Stamatovic et al., 2016). Previous experiments have demonstrated that decreasing ZO-1, occludin, and claudin-5 expression could increase the BTB permeability (Cai et al., 2015b; Ma et al., 2016).

EMAP-II is a multifunctional polypeptide that functions as a proinflammatory cytokine, and has been studied in antitumor and antiangiogenic activities (Awasthi et al., 2013; Mogylnytska, 2015). EMAP-II consists of 169 amino acid residues and exerts an important role, as a cytokine, in inflammation, apoptosis and angiogenesis (Lozhko et al., 2013; Liu et al., 2015). Studies on ECs have shown that EMAP-II has a biological role in anti-angiogenesis (Liu et al., 2015). In a study of glioma cells in nude mice, EMAP-II showed specific toxicity *in vitro* against GECs and, as an anti-angiogenic therapy, it stopped glioma growth as a tumor blood vessel inhibitor and by VEGF inhibition (Schwarz and Schwarz, 2004). Previous studies have shown that EMAP-II can open the BTB by activating RhoA/ROCK/PI3K (Li Z. et al., 2015), cAMP/PKA/Rac1 (Li et al., 2016b), RhoA/Rho kinase/PKC-α/β/PP1 (Li et al., 2016a) and other signaling pathways (Cai et al., 2015a). However, whether miRNAs, as emerging regulators of signal transduction, are involved in EMAP-II regulation of the BTB permeability is still unknown.

miR-429, as a member of the miR-200 family, has been demonstrated to be one of the major regulatory molecules of the EMT (Park et al., 2008). Most studies have indicated that miR-429 plays a role in tumor suppression to prevent cancer formation in healthy cells. miR-429 expression is down-regulated in non-small cell lung cancer (Xiao et al., 2016), colorectal cancer (Dong et al., 2016), renal cell carcinoma (Chen et al., 2016) and esophageal cancer (Bartoszewska et al., 2015). Up-regulation of miR-429 inhibits the ability of tumor cells to proliferate, migrate and invade (Pecot et al., 2013; Wang et al., 2013; Yoshino et al., 2013; Zhu et al., 2014). In addition, miR-429 was shown to decreased glioma invasion by targeting BMK1 (Chen et al., 2015). However, it has not been fully established if miR-429 can regulate the permeability of the BTB.

P70S6K, as a downstream effector of the PI3K/Akt/mTOR signal transduction pathway, is a member of the serine/threonine protein kinase family, (Zanchi and Lancha, 2008; Zhao and Gartenhaus, 2009). P70S6K phosphorylates ribosomal S6 protein, which are both engaged in various cellular functions, such as proliferation, tumorigenesis, angiogenesis, differentiation and apoptosis (Zhao and Gartenhaus, 2009). There are many studies concerning the role of p70S6K in regulating biological behavior in gliomas. By inhibiting the mTOR/p70S6K1 signaling pathway, GSK-3β was shown to decrease glioma progression *in vivo* (Zhao et al., 2015). Furthermore, FTY720-induced inhibition of the PI3K/AKT/mTOR/p70S6K signaling pathway was shown to suppress migration and invasion in human glioblastoma cell lines (Zhang et al., 2014b). In addition, cathepsin S-induced inhibition of the PI3K/AKT/mTOR/p70S6K signaling pathway was found to cause autophagy in human glioblastoma cell lines (Zhang et al., 2014a). However, there are no relevant studies which report that p70S6K can regulate TJ-associated protein expression and influence the BTB permeability in gliomas. This study aimed to investigate whether EMAP-II could influence TJ-associated protein expression and change the BTB permeability by modulating miR-429 expression and thus directly or indirectly regulating p70S6K.

## Materials and Methods

### Cell Lines and Culture

The immortalized human cerebral micro vascular EC line hCMEC/D3 (provided by Dr. Pierre-Olivier Couraud, Institut Cochin, France) was cultured according to Weksler et al. (2005). Endothelial basal medium-2 (EBM-2, Lonza, Walkersville, MD, United States), supplemented with 5% fetal bovine serum (FBS, PAA Laboratories, Pasching, Austria), 10 mM HEPES (PAA Laboratories, Pasching, Austria), 1% Penicillin–Streptomycin (Life Technologies), 1% chemically defined lipid concentrate (Life Technologies Corporation, Paisley, United Kingdom), 1.4 mM hydrocortisone (Sigma–Aldrich, St. Louis, MO, United States), 5 mg/ml ascorbic acid (Sigma–Aldrich) and 1 ng/ml human basic fibroblast growth factor (bFGF) (Sigma–Aldrich, St. Louis, MO, United States). Cultrex Rat Collagen-I (R&D Systems, Minneapolis, MN, United States) was previously coated onto the culture dishes before cells had been seeded. Human glioblastoma U87 cell line and human embryonic kidney 293T cell line were got from Cell Bank of Chinese Academy of Sciences and maintained in Dulbecco’s modified Eagle’s medium (DMEM) of high glucose supplemented with 10% FBS, 100 U/ml penicillin, and 100 μg/ml streptomycin (Life Technologies). All the cells were cultured at 37°C with 5% CO_2_ in the humid atmosphere, and the medium was changed every 48 or 72 h. EMAP-II was purchased from PeproTech (Rehovot, Israel).

### Establishment of BTB and BBB Models *in Vitro*

*In vitro* BTB models were established following the procedure as above (Nakagawa et al., 2007; Cai et al., 2015a). Tanswell systems (0.4-μm pore size; Corning, NY, United States) were applied to co-culture the U87 and hCMEC/D3 cells, and U87 cells were seeded (2 × 10^4^/well) in six-well plates and cultured for 24 h. Then hCMEC/D3 cells were seeded (2 × 10^5^/well) on the upper side of transwell’s filter membranes pre-coated with 150 μg/ml Cultrex Rat Collagen-I. After co-cultured for 96 h, the GECs from *in vitro* BTB models were obtained for further analysis. For the BBB models, normal human astrocytes were seeded instead of U87 cells. In the following experiment EMAP-II was added to the upper chamber which made hCMEC/D3 cells were directly exposed to the EMAP-II. EMAP-II was exposured to the apical cell surface of the endothelia.

### Cells Transfection and Grouping

The miR-429 overexpression and miR-429 silencing plasmids were constructed separately in pGPH1/GFP/Neo (pre-miR-429) and pGPU6/GFP/Neo (anti-miR-429) (GenePharma, Shanghai, China). pGCMV/MCS/T2A/EGFP/Neo [p70S6K(+)] and pGPU6/GFP/Neo [p70S6K(-)] (GenePharma, Shanghai, China) were DNA plasmid vectors. Both of them were stably transfected and cells were selected by the culture medium containing 0.4 mg/mL G418 (Sigma–Aldrich) in the following 3 or 4 weeks. The transfected efficiencies were assessed by quantitative Real-time PCR (qRT-PCR) and western blot analysis. To get the cotransfection cells for further study, miR-429 agomir, miR-429 antagomir, and negative controls were, respectively, transfected transiently into p70S6K stably transfected cells according to the instructions of Lipofectamine 2000 (Life Technologies Corporation, Carlsbad, CA, United States). Cotransfected cells were got from the culture in 48 h and grouped into nine (**Table 1**).

**Table 1:** Grouping the co-transfected cells.

Group	Stable transfected cells	Co-transfection
Control	untransfected	none
pre-miR-429-NC+p70S6K(+)-NC	p70S6K(+)-NC	pre-miR-429-NC
pre-miR-429+p70S6K(+)	p70S6K(+)	pre-miR-429
pre-miR-429-NC+p70S6K(-)-NC	p70S6K(-)-NC	pre-miR-429-NC
pre-miR-429+p70S6K(-)	p70S6K(-)	pre-miR-429
anti-miR-429-NC+p70S6K(+)-NC	p70S6K(+)-NC	anti-miR-429-NC
anti-miR-429+p70S6K(+)	p70S6K(+)	anti-miR-429
anti-miR-429-NC+p70S6K(-)-NC	p70S6K(-)-NC	anti-miR-429-NC
anti-miR-429+p70S6K(-)	p70S6K(-)	anti-miR-429

### TEER Values and HRP Flux

After BTB models were established, the TEER assays were performed by millicell-ERS instrument (Millipore, Billerica, MA, United States). Based on our previous experiments (Cai et al., 2015a), in 96 h of co-culture, TEER values were measured. Before final resistances were calculated, background electrical resistances had been subtracted. The final TEER values were measured as ohms per square centimeter. After BTB models were constructed, 1 ml serum-free EBM-2 containing 0.5 μmol/L HRP was added into the upper compartment of the transwell. In 1 h, the medium in the lower compartment was collected and TMB colorimetry was applied to test the collected samples by spectrophotometer at 370 nm. The HRP flux was shown as picomoles passed per square centimeter surface area per hour (pmol/cm^2^/h).

### Laser Capture Microdissection (LCM) and Quantitative Real-Time PCR (qRT-PCR)

Laser capture microdissection (LCM) was performed following previously description (Cai et al., 2015b). The specimens were fresh-frozen sectioned with 10 μm thickness by Microtome Cryostat (MICROM International GmbH, Walldorf, Germany) and then stained by Ulex europaeus agglutinin I (UEA-I) (Vector Laboratories, Burlington, ON, Canada) to mark the target vessels. ArcturusXT^TM^ instrument (Applied Biosystems, Foster City, CA, United States) was applied to perform precision cutting. The GECs or ECs were captured and transferred onto CapSure^®^ HS LCM Caps (Invitrogen, United States). Parameters were set as below: 7.5 μm for laser spot size, 50 mV for power and 0.7 ms for duration time.

Total RNA was extracted by Trizol reagent (Life Technologies Corporation, Carlsbad, CA, United States). RNA concentration and quality were measured with the 260/280 nm ratio by a Nanodrop Spectrophotometer (ND-100; Nano Drop, Wilmington, DE, United States). Real-time PCR was done to test the expression levels of miR-429 and p70S6K by 7500 Fast Real-Time PCR System (Applied Biosystems). Taqman MicroRNA Reverse Transcription Kit and Taqman Universal Master Mix II were used in the TaqMan MicroRNA assays of miR-429 and U6 (Applied Biosystems, Foster City, CA, United States). The High Capacity cDNA Reverse Transcription Kits and TaqMan Universal Master Mix II were used in the gene expression assays of p70S6K and GAPDH (Applied Biosystems, Foster City, CA, United States). Fold changes were calculated by standard (2^-ΔΔCt^) method.

### Western Blot

The protein concentrations were set by the BCA protein assay kit (Beyotime Institute of Biotechnology). All cell lysates with 40 μg proteins were loaded onto SDS-polyacrylamide gels and blotted onto polyvinylidene difluoride membranes. The membranes were blocked by 5% nonfat dry milk in TBST for 2 h and then incubated overnight at 4°C with primary antibodies against p70S6K, S6K(1:500; Proteintech, Chicago, IL, United States), p- p70S6K, p-S6K (1:200; Santa Cruz Biotechnology, Santa Cruz, CA, United States), ZO-1 (1:500; Life Technologies), occludin (1:250; Life Technologies), claudin-5 (1:500; Life Technologies) and GAPDH (1:10,000; Proteintech). After washed for three times by the PBS-Tween, the membranes were incubated in the secondary antibody diluted at 1:5000 at room temperature for 2 h. Protein bands were visualized by enhanced chemiluminescence (Santa Cruz Biotechnology, Santa Cruz, CA, United States) and measured by the ECL Detection System (Thermo Scientific, United States). The protein bands were scanned by Chemi Imager 5500 V2.03 software, and integrated light density values were tested by Fluor Chen 2.0 software. GAPDH was used to normalize the above results.

### Clinical Specimens

Glioma tissues and NBTs were got from the Department of Neurosurgery, Shengjing Hospital of China Medical University from September 2013 to July 2014. Low grade gliomas (LGGs) are mainly brain tumors classified as grade I and II by the WHO grading system and high grade gliomas (HGGs) are grade III and IV. After surgical resection, the specimens were immediately frozen and preserved in liquid nitrogen. All participants provided written consent and the procedures were approved by the Ethics Committee at the Shengjing Hospital of China Medical University.

### Dual-Luciferase Reporter Assay

TargetScan Human Release 6.2^1^ predicted there was one potential binding site between the 3′UTR of ZO-1 mRNA and the seed region of miR-429. To examine whether miR-429 targets ZO-1 directly, wild-type ZO-1 3′UTR reporter plasmids (ZO-1 Wt) and mutated-type ZO-1 3′UTR reporter plasmids (ZO-1 Mut) were constructed in the pmirGLO-promoter vector. HEK 293T cells were cultured in 96-well microtiter plates (Corning, NY, United States) for 24 h. MiR-429 overexpressed and NC plasmids were transfected with ZO-1 Wt 3′UTR and ZO-1 Mut 3′UTR, respectively, by Lipofectamine 2000. In 48 h of transfection, the luciferase activities were tested by the Dual-Luciferase Reporter Assay System (Promega, Madison, WI, United States). Luciferase expressions were shown as related light units (firefly/Renilla luciferase) to determine whether ZO-1 was the target of miR-429 *in vitro*. Based on two potential binding sites of the miR-429 and occludin, Mut 1, Mut 2, and Mut 3 were constructed. Based on one potential binding site of the miR-429 and p70S6K, Mut was constructed.

### Immunofluorescence Assays

The GECs on insert filters were fixed in 4% paraformaldehyde for 20 min and blocked by 5% BSA for 2 h at room temperature. After washed with PBS for 3 times, GECs were incubated by primary antibodies of ZO-1, occludin, and claudin-5 (1:50; Life Technologies) at 4°C overnight. The nuclei were stained by DAPI (0.5 mg/ml, Beyotime Institute of Biotechnology) for 8 min. The fluorescence was visualized by DP71 immunofluorescence microscope (Olympus, Tokyo, Japan), and merged by Chemi Imager 5500 V2.03 software.

### Statistical Analysis

All data were showed as mean ± SD for every group and analyzed by SPSS 21 software. Differences between two groups were tested for significance with the Student’s *t*-test. One-way analysis of variance and Dunnett’s post-test were used to know the significance among groups. The values were considered to be significant when *P* < 0.05.

## Results

### EMAP-II Promoted BTB Permeability in a Time-and-Dose Dependent Pattern and Down-Regulated ZO-1, Occludin and Claudin-5 in GECs of BTB Models

**FIGURE 1:**
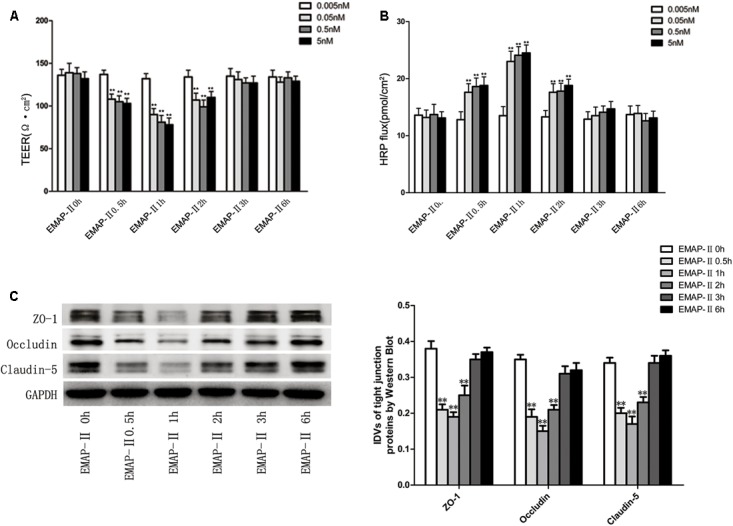
The integrity and permeability of BTB with the EMAP-II influence. **(A)** TEER values of BTB models were shown as Ω•cm^2^. All data represent mean ± SD (*n* = 5, each). ^∗∗^*P* < 0.01 vs. EMAP-II 0 h groups. **(B)** Permeability assays were performed by HRP fluxes (45 kDa). HRP fluxes were calculated as pmol/cm^2^/h. All data represent mean ± SD (*n* = 5, each). ^∗∗^*P* < 0.01 vs. EMAP-II 0 h groups. **(C)** The protein expression levels of ZO-1, occludin and claudin-5 with the EMAP-II influence were tested by western blot. All data represent mean ± SD (*n* = 5, each). ^∗∗^*P* < 0.01 vs. EMAP-II 0 h groups.

Based on the results of pre-experiments, after the successful establishment of *in vitro* BTB models, EMAP-II with different concentrations of 0.005, 0.05, 0.5, and 5 nM were used to influence BTB models, respectively. TEER values and HRP flux were tested and the changes of BTB models permeability were evaluated in 0.5, 1, 2, 3, and 6 h. The results showed that there was no significant difference of TEER values in the 0.005 nM group. For 0.05, 0.5, and 5 nM groups, the TEER values influenced by EMAP-II were significantly lower than those of the control groups in 0.5, 1, and 2 h, of which the most significant was in 1 h. Meanwhile in 3 and 6 h, there was no statistical difference (**Figure 1A**). There was no significant difference in the HRP flux in the 0.005 nM group. As for 0.05, 0.5, and 5 nM groups, the HRP flux influenced by EMAP-II in 0.5, 1, and 2 h were greatly higher than those of the control groups, of which the most significant was in 1 h. Meanwhile in 3 and 6 h, there was no statistical difference (**Figure 1B**). Based on the above experimental results, EMAP-II with concentration of 0.05 nM was chosen to carry out the following experiments. EMAP-II of 0.05 nM was used to influence BTB models; and in 0.5, 1, 2, 3, and 6 h, western blot was applied to detect changes of TJ-associated proteins (ZO-1, occludin and claudin-5) in human GECs. Results showed that with the EMAP-II influence, the protein expression of ZO-1, occludin and claudin-5 was greatly lower than that of the control groups in 0.5, 1, and 2 h, of which the most significant was in 1 h. Meanwhile there was no statistical difference in 3 and 6 h (**Figure 1C**).

### The Expressions of miR-429 in GECs and Glioma Tissues, and the Effect of EMAP-II on miR-429 Expressions

**FIGURE 2:**
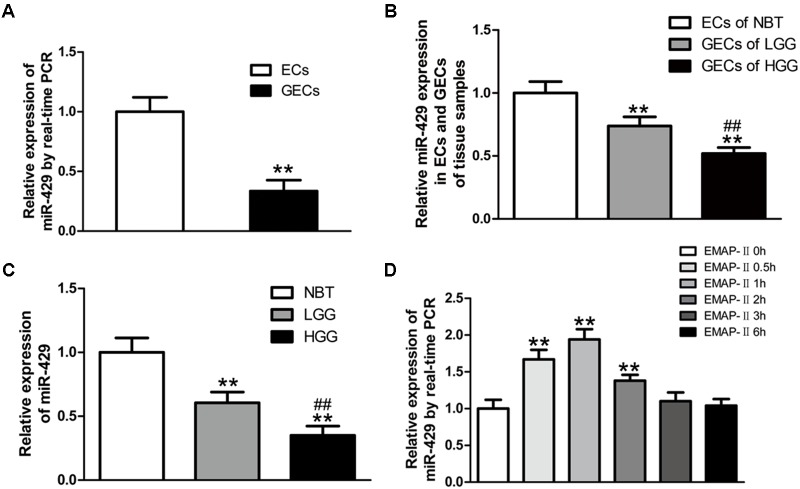
The variety of miR-429 expression and the effect of EMAP-II on the expression of miR-429. **(A)** The relative expressions of miR-429 were tested in ECs and GECs from cell lines by qRT-PCR. All data represent mean ± SD (*n* = 5, each). ^∗∗^*P* < 0.01 vs. ECs groups. **(B)** The relative expressions of miR-429 were tested in ECs of NBT, GECs of LGG, and GECs of HGG. All data represent mean ± SD (*n* = 5, each). ^∗∗^*P* < 0.01 vs. NBT groups, ^##^*P* < 0.01 vs. LGG groups. **(C)** The relative expressions of miR-429 were tested in NBT, LGG and HGG. All data represent mean ± SD (*n* = 5, each). ^∗∗^*P* < 0.01 vs. NBT groups, ^##^*P* < 0.01 vs. LGG groups. **(D)** The relative expressions of miR-429 in GECs when treated by EMAP-II were tested by qRT-PCR. All data represent mean ± SD (*n* = 5, each). ^∗∗^*P* < 0.01 vs. EMAP-II 0 h groups.

*In vitro* BBB and BTB models were established, and miR-429 expression was tested by qRT-PCR in human ECs and GECs. The result showed that miR-429 expression greatly decreased in human GECs compared with those in ECs (**Figure 2A**). LCM assay was used to collect the ECs of NBT and GECs of glioma tissues. The expression of miR-429 was tested by qRT-PCR in ECs of NBT, GECs of LGG, and GECs of HGG. The result showed that miR-429 expression in GECs of LGG and GECs of HGG decreased significantly compared with those in ECs of NBT, and miR-429 expression in GECs of HGG decreased significantly compared with those in GECs of LGG (**Figure 2B**). The endogenous expression level of miR-429 was measured in NBT, LGG, and HGG. The result showed that miR-429 expression of LGG and HGG decreased significantly compared with those of NBT, and miR-429 expression of HGG decreased significantly compared with those of LGG (**Figure 2C**). After EMAP-II had been used in BTB models for 0.5, 1, 2, 3, and 6 h, qRT-PCR was used to test miR-429 expression in GECs. Result showed that miR-429 expression significantly elevated in 0.5, 1, and 2 h compared with the control groups, of which the most significant was in 1 h and there was no statistical difference in 3 and 6 h (**Figure 2D**).

### MiR-429 Overexpression and miR-429 Silencing Significantly Changed the BTB Permeability

**FIGURE 3:**
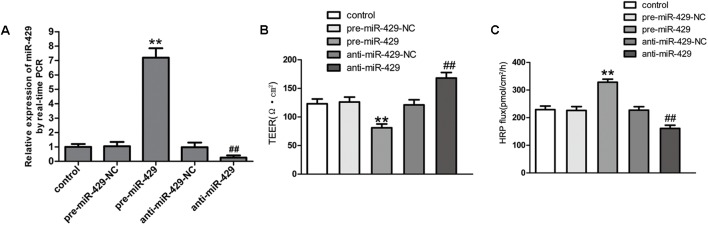
miR-429 regulating BTB permeability. **(A)** The relative miR-429 expressions of miR-429 overexpression and silencing. All data represent mean ± SD (*n* = 5, each). ^∗∗^*P* < 0.01 vs. pre-miR-429-NC groups. ^##^*P* < 0.01 vs. anti-miR-429-NC groups. **(B)** TEER values of BTB models were shown as Ω•cm^2^. All data represent mean ± SD (*n* = 5, each). ^∗∗^*P* < 0.01 vs. pre-miR-429-NC groups, ^##^*P* < 0.01 vs. anti-miR-429-NC groups. **(C)** Permeability assays were performed by HRP fluxes (45 kDa). HRP fluxes were calculated as pmol/cm^2^/h. All data represent mean ± SD (*n* = 5, each). ^∗∗^*P* < 0.01 vs. pre-miR-429-NC groups, ^##^*P* < 0.01 vs. anti-miR-429-NC groups.

To further study the effect of miR-429 on BTB permeability, miR-429 plasmids of the overexpression and silencing expression, and corresponding negative control plasmids were transfected into hCMEC/D3 cells. After that, G418 was used to select the stably transfected cells. qRT-PCR assay was applied to test miR-429 expression in stably transfected cells. The result showed miR-429 expression greatly increased in pre-miR-429 group compared with pre-miR-429-NC group; miR-429 expression significantly decreased in anti-miR-429 group compared with anti-miR-429-NC group (**Figure 3A**). After the overexpression and silencing miR-429 BTB models were successfully established, TEER values and HRP flux were performed, respectively. It was found that TEER value of pre-miR-429 group was greatly lower than that in pre-miR-429-NC group; TEER value of anti-miR-429 group was greatly higher than those in anti-miR-429-NC groups (**Figure 3B**). HRP fluxes in pre-miR-429 group was greatly higher than those in pre-miR-429-NC group; HRP fluxes in anti-miR-429 group was greatly lower than those in anti-miR-429-NC group (**Figure 3C**).

### MiR-429 Regulated the Expressions of TJ-Associated Proteins and ZO-1 and Occludin Were Direct Targets of miR-429

**FIGURE 4:**
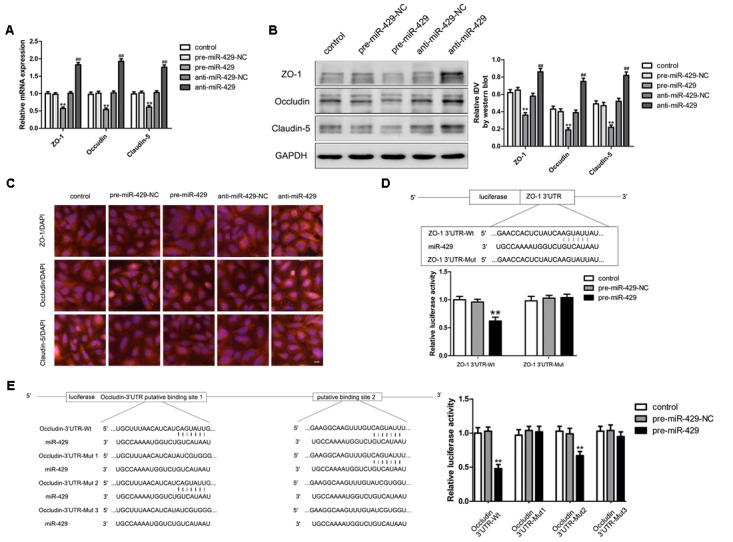
miR-429 influencing the expression of ZO-1, occludin and claudin-5. **(A)** The mRNA expressions of ZO-1, occludin and claudin-5 were tested by qRT-PCR of miR-429 overexpression and silencing. All data represent mean ± SD (*n* = 5, each). ^∗∗^*P* < 0.01 vs. pre-miR-429-NC groups, ^##^*P* < 0.01 vs. anti-miR-429-NC groups. **(B)** The proteins expressions of ZO-1, occludin and claudin-5 were tested by western blot of miR-429 overexpression and silencing. All data represent mean ± SD (*n* = 5, each). ^∗∗^*P* < 0.01 vs. pre-miR-429-NC groups, ^##^*P* < 0.01 vs. anti-miR-429-NC groups. **(C)** The expressions and distributions of ZO-1, occludin and claudin-5 were tested by immunofluorescence. (*n* = 5, scale bar = 5 μm). ZO-1 **(D)** and occludin **(E)** were the target genes of miR-429. Relative luciferase activities were shown as firefly/renilla luciferase activities. All data represent mean ± SD (*n* = 5, each). ^∗∗^*P* < 0.01 vs. ZO-1/occludin 3′UTR Wt+pre-miR-429-NC groups.

qRT-PCR and western blot were applied to test the mRNA and protein expressions of TJ-associated proteins (ZO-1, occludin and claudin-5) *in vitro* BTB models. The results showed that the expressions of ZO-1, occludin and claudin-5 in pre-miR-429 groups were remarkably lower than those in pre-miR-429-NC groups; the expressions of ZO-1, occludin and claudin-5 in anti-miR-429 groups were remarkably higher than those in anti-miR-429-NC groups (**Figures 4A,B**). Immunofluorescence was applied to test the expressions and distributions of TJ-associated proteins (ZO-1, occludin and claudin-5) *in vitro* BTB models. The results showed ZO-1, occludin and claudin-5 were located on the edge of GECs with the continuous distribution in control, pre-miR-429-NC and anti-miR-429-NC groups. The fluorescence intensity of pre-miR-429 groups weakened into a discontinuous distribution compared with pre-miR-429-NC groups; whereas the fluorescence intensity of anti-miR-429 groups enhanced compared with anti-miR-429-NC groups (**Figure 4C**).

Bioinformatics software Human Release TargetScan 6.2^2^ was used to test the potential targets of miR-429; and the result showed that there was a potential binding site at 71–77 of ZO-1 mRNA 3′UTR, and the site sequence and the miR-429 seed regions are complementary and matched to each other. Dual luciferase reporter gene assay showed that the relative luciferase activities of ZO-1 3′UTR-Wt+pre-miR-429 groups were significantly inhibited compared with ZO-1 3′UTR-Wt+pre-miR-429-NC groups; whereas the relative luciferase activities of ZO-1 3′UTR-Mut+pre-miR-429 groups had no significant changes compared with ZO-1 3′UTR-Wt+pre-miR-429-NC groups. The results proved that miR-429 targeted the 3′UTR of ZO-1 (**Figure 4D**). Bioinformatics software Human Release TargetScan 6.2^2^ was applied to test the potential targets of miR-429, and the results showed that there were two potential binding sites at 139–145 and 2151–2157 of occludin mRNA 3′UTR. The above sites sequence and the miR-429 seed region are complementary and matched to each other. Mut1 was the single mutant at 139–145, Mut2 was the single mutant at 2151–2157, and Mut3 was simultaneous mutants at both 139–145 and 2151–2157. Dual luciferase reporter gene assays showed that the relative luciferase activities of occludin 3′UTR-Wt+pre-miR-429 groups were significantly inhibited compared with occludin 3′UTR-Wt+pre-miR-429-NC groups; the related luciferase activities of occludin 3′UTR-Mut1+pre-miR-429 groups had no significant changes compared with occludin 3′UTR-Mut1+pre-miR-429-NC groups; the related luciferase activities of occludin 3′UTR-Mut2+pre-miR-429 groups were significantly inhibited compared with occludin 3′UTR-Mut2+pre-miR-429-NC groups; the related luciferase activities of occludin 3′UTR-Mut3+pre-miR-429 groups had no significant changes compared with occludin 3′UTR-Mut3+pre-miR-429-NC groups (**Figure 4E**). The results pointed out miR-429 and occludin mRNA3′UTR combined at sequence 139–145.

### EMAP-II Significantly Reduced the p70S6K Expression in GECs of BTB Models, and p70S6K Changed the BTB Permeability and TJ-Associated Proteins Expressions

**FIGURE 5:**
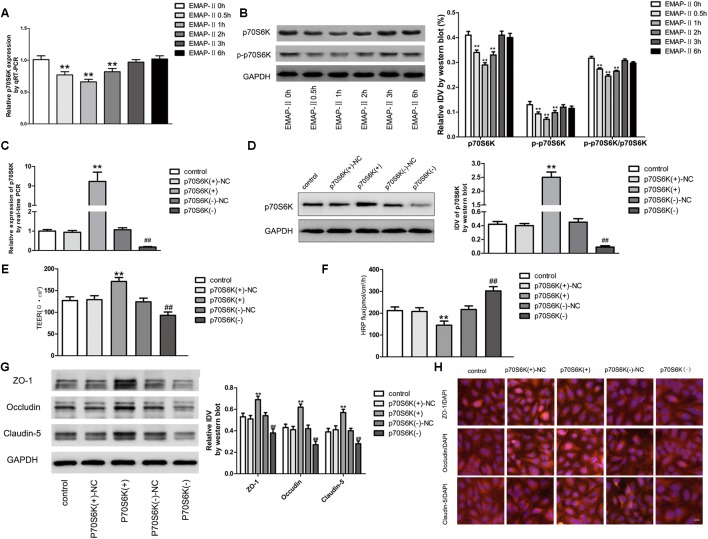
p70S6K influencing BTB permeability and the expression of ZO-1, occludin and claudin-5. **(A)** The mRNA expressions of p70S6K with the EMAP-II influence by qRT-PCR. All data represent mean ± SD (*n* = 5, each). ^∗∗^*P* < 0.01 vs. EMAP-II 0 h groups. **(B)** The protein expressions of p70S6K with the EMAP-II influence by western blot. All data represent mean ± SD (*n* = 5, each). ^∗∗^*P* < 0.01 vs. EMAP-II 0 h groups. The relative p70S6K expressions of p70S6K overexpression and silencing were tested by qRT-PCR **(C)** and western blot **(D)**. All data represent mean ± SD (*n* = 5, each). ^∗∗^*P* < 0.01 vs. p70S6K(+)–NC groups; ^##^*P* < 0.01 vs. p70S6K(–)–NC groups. The integrity and permeability of BTB models of p70S6K overexpression and silencing were tested. **(E)** TEER values of BTB models were shown as Ω•cm^2^. All data represent mean ± SD (*n* = 5, each). ^∗∗^*P* < 0.01 vs. p70S6K (+)–NC groups, ^##^*P* < 0.01 vs. p70S6K (–)– NC groups. **(F)** Permeability assays were performed by HRP fluxes (45 kDa). HRP fluxes were calculated as pmol/cm^2^/h. All data represent mean ± SD (*n* = 5, each). ^∗∗^*P* < 0.01 vs. p70S6K (+)–NC groups, ^##^*P* < 0.01 vs. p70S6K(–)– NC groups. The proteins expressions and distributions of ZO-1, occludin and claudin-5 of p70S6K overexpression and silencing. **(G)** The proteins expressions of ZO-1, occludin and claudin-5 were tested by western blot. All data represent mean ± SD (*n* = 5, each). ^∗∗^*P* < 0.01 vs. p70S6K (+)–NC groups, ^##^*P* < 0.01 vs. p70S6K (–)–NC groups. **(H)** The expressions and distributions of ZO-1, occludin and claudin-5 were tested by Immunofluorescence. (*n* = 5, scale bar = 5 μm).

For EMAP-II of 0.05 nM in BTB models in 0.5, 1, 2, 3, and 6 h, qRT-PCR and western blot were used to test p70S6K mRNA and protein expressions in GECs, and the protein expression of p-p70S6K. The results showed that in 0.5, 1, and 2 h, p70S6K mRNA and protein expressions were significantly lower than those in control groups, of which the most significant was in 1 h. With time passing by, the effect gradually weakened and there was no statistical difference in 3 and 6 h. It had the same trend in p-p70S6K and p-p70S6K/p70S6K (**Figures 5A,B**). Plasmids of p70S6K overexpression and silencing, and corresponding negative control plasmids were transfected into hCMEC/D3 cells separately; and G418 was used to screen the stable cells. Transfection efficiency was verified by qRT-PCR and western blot (**Figures 5C,D**). After the establishment of p70S6K overexpression and silencing *in vitro* BTB models, the TEER values and HRP fluxes in BTB models were performed. Results showed that the TEER values of p70S6K(+) groups were significantly higher than those of p70S6K(+)-NC groups and p70S6K(-) groups had the opposite results (**Figure 5E**). HRP fluxes of p70S6K(+) groups were significantly lower than those of p70S6K(+)-NC groups; HRP fluxes of p70S6K(-) groups were significantly higher than those of p70S6K(-)-NC groups (**Figure 5F**).

Western blot was applied to test the expressions of TJ-associated proteins (ZO-1, occludin and claudin-5) of GECs *in vitro* BTB models with p70S6K overexpression and silencing, respectively. Results showed that the protein expressions of ZO-1, occludin and claudin-5 in p70S6K(+) groups were significantly higher than those in p70S6K(+)-NC groups, and the protein expressions of ZO-1, occludin and claudin-5 in p70S6K(-) groups were significantly lower than those in p70S6K(-)-NC groups (**Figure 5G**). The immunofluorescence was applied to detect the expressions and distribution of ZO-1, occludin and claudin-5 *in vitro* BTB models with p70S6K overexpression and silencing, respectively. Results showed that ZO-1, occludin and claudin-5 were located on the edge of GECs, which were continuously distributed; the fluorescence intensity of p70S6K(+) groups enhanced, and the distribution became more continuous compared with p70S6K(+)-NC groups; whereas the fluorescence intensity of p70S6K(-) groups reduced and the distributions became less continuous compared with p70S6K(-)-NC groups (**Figure 5H**).

### MiR-429 Negatively Regulated the Expressions of p70S6K by Direct Targeting

**FIGURE 6:**
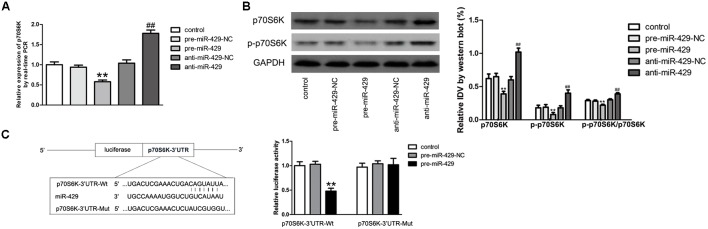
miR-429 regulating p70S6K. **(A)** The mRNA expressions of p70S6K of miR-429 overexpression and silencing were tested by qRT-PCR. All data represent mean ± SD (*n* = 5, each). ^∗∗^*P* < 0.01 vs. pre-miR-429-NC groups, ^##^*P* < 0.01 vs. anti-miR-429-NC groups. **(B)** The p70S6K protein expressions of miR-429 overexpression and silencing were tested by western blot. All data represent mean ± SD (*n* = 5, each). ^∗∗^*P* < 0.01 vs. pre-miR-429-NC groups, ^##^*P* < 0.01 vs. anti-miR-429-NC groups. **(C)** P70S6K was the target gene of miR-429. Relative luciferase activities were shown as firefly/renilla luciferase activities. All data represent mean ± SD (*n* = 5, each). ^∗∗^*P* < 0.01 vs. p70S6K Wt+pre-miR-429-NC groups.

qRT-PCR and western blot were applied to detect the expressions of p70S6K *in vitro* BTB models of miR-429 overexpression and silencing. Results showed that p70S6K mRNA and protein expressions of pre-miR-429 groups were remarkably lower than those of pre-miR-429-NC groups, p70S6K mRNA and protein expressions of anti-miR-429 were remarkably higher than those of anti-miR-429-NC groups (**Figures 6A,B**). Bioinformatics software Human Release TargetScan 6.2^3^ was applied to predict the potential targets of miR-429. Result showed that there was one potential miR-429 binding site at 291–298 of p70S6K mRNA 3′UTR. The above site sequence and the miR-429 seed region were complementary and matched to each other. Dual luciferase reporter gene analysis revealed the relative luciferase activities were significantly inhibited in the p70S6K 3′UTR- Wt+pre-miR-429 compared with p70S6K 3′UTR-Wt+pre-miR-429-NC; whereas compared with p70S6K 3′UTR-Mut+pre-miR-429-NC, there was no significant changes of the relative luciferase activities in p70S6K 3′UTR-Mut+pre-miR-429 (**Figure 6C**). The result proved that miR-429 targeted the 3′UTR of p70S6K.

### Cotransfection of miR-429 and p70S6K Reversely Changed the BTB Permeability, and miR-429 Regulated ZO-1, Occludin and Claudin-5 Expressions by Affecting p70S6K-S6 Signaling Pathway

**FIGURE 7:**
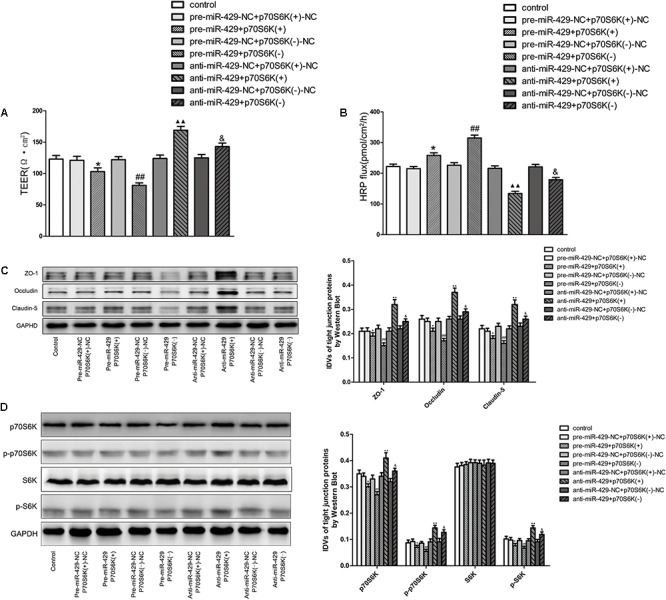
p70S6K involved in miR-429 regulating BTB permeability and the expression of ZO-1, occludin and claudin-5. The TEER values **(A)** and HRP fluxes **(B)** of BTB models were performed. TEER values were shown as Ω•cm^2^. HRP fluxes were calculated as pmol/cm^2^/h. All data represent mean ± SD (*n* = 5, each). ^∗^*P* < 0.05 vs. pre-miR-429-NC+p70S6K(+)–NC groups, ^##^*P* < 0.01 vs. pre-miR-429-N+p70S6K(–)–NC groups. ^
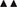
^*P* < 0.01 vs. anti-miR-429-NC+p70S6K(+)–NC groups. ^&^*P* < 0.05 vs. anti -miR-429-NC+p70S6K(–)–NC groups. **(C)** Western blot was used to test the proteins expressions of ZO-1, occludin and claudin-5 in GECs which had been reversely co-regulated by miR-429 and p70S6K. All data represent mean ± SD (*n* = 5, each). ^∗^*P* < 0.05 vs. pre-miR-429-NC+p70S6K(+)–NC groups, ^##^*P* < 0.01 vs. pre-miR-429-NC+p70S6K(–)–NC groups. ^
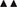
^*P* < 0.01 vs. anti-miR-429-NC+p70S6K(+)–NC groups. ^&^*P* < 0.05 vs. anti-miR-429-NC+p70S6K(–)–NC groups. **(D)** The proteins expressions of p70S6K, p-p70S6K, S6K, and p-S6K were reversely co-regulated by miR-429 and p70S6K. All data represent mean ± SD (*n* = 5, each). ^∗^*P* < 0.05 vs. pre-miR-429-NC+p70S6K(+)–NC groups, ^##^*P* < 0.01 vs. pre-miR-429-NC+p70S6K(–)–NC groups. ^
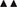
^*P* < 0.01 vs. anti-miR-429-NC+p70S6K(+)–NC groups. ^&^*P* < 0.05 vs. anti-miR-429-NC+p70S6K(–)–NC groups.

To study the role of p70S6K in miR-429 mediating the regulation of BTB permeability, cotransfection of miR-429 and p70S6K *in vitro* BTB models was established. TEER values and HRP flux assays were used to test the BTB permeability. Results showed that TEER values of pre-miR-429+p70S6K(+) groups decreased compared with pre-miR-429-NC+p70S6K(+)-NC groups, TEER values of pre-miR-429+p70S6K(-) groups remarkably decreased compared with pre-miR-429-NC+p70S6K(-)-NC groups, TEER values of anti-miR-429+p70S6K(+) groups remarkably increased compared with anti-miR-429-NC+p70S6K(+)-NC groups, and TEER values of anti-miR-429+p70S6K(-) groups increased compared with anti-miR-429-NC+p70S6K(-)-NC groups (**Figure 7A**). HRP fluxes of pre-miR-429+p70S6K(+) groups increased compared with pre-miR-429-NC+p70S6K(+)-NC groups, HRP fluxes of pre-miR-429+p70S6K(-) groups remarkably increased compared with pre-miR-429-NC+p70S6K(-)-NC groups, HRP fluxes of anti-miR-429+p70S6K(+) groups remarkably decreased compared with anti-miR-429-NC+p70S6K(+)-NC groups, and HRP fluxes of anti-miR-429+p70S6K(-) groups decreased compared with anti-miR-429-NC+p70S6K(-)-NC groups (**Figure 7B**). The above results proved that cotransfection of miR-429 and p70S6K reversely regulated the BTB permeability.

Western blot was used to test the protein expressions of ZO-1, occludin and claudin-5 *in vitro* BTB models with cotransfection of miR-429 and p70S6K. Results showed that the expressions of ZO-1, occludin and claudin-5 in pre-miR-429+p70S6K(+) groups decreased compared with pre-miR-429-NC+p70S6K(+)-NC groups, the expressions of ZO-1, occludin and claudin-5 in pre-miR-429+p70S6K(-) groups remarkably decreased compared with pre-miR-429-NC+p70S6K(-)-NC groups, the expressions of ZO-1, occludin and claudin-5 in anti-miR-429+p70S6K(+) groups remarkably increased compared with anti-miR-429-NC+p70S6K(+)-NC groups and the expressions of ZO-1, occludin and claudin-5 in anti-miR-429+p70S6K(-) groups increased compared with anti-miR-429-NC+p70S6K(-)-NC groups (**Figure 7C**). The above results proved that cotransfection of miR-429 and p70S6K reversely regulated the expression of ZO-1, occludin and claudin-5.

Western blot was used to detect the protein expressions of p70S6K, p-p70S6K, S6K, and p-S6K *in vitro* BTB models with cotransfection of miR-429 and p70S6K. Results showed that the expressions of p70S6K, p-p70S6K, and p-S6K in pre-miR-429+p70S6K(+) groups decreased compared with pre-miR-429-NC+p70S6K(+)-NC groups, the expressions of p70S6K, p-p70S6K, and p-S6K in pre-miR-429+p70S6K(-) groups remarkably decreased compared with pre-miR-429-NC+p70S6K(-)-NC groups, the expressions of p70S6K, p-p70S6K, and p-S6K in anti-miR-429+p70S6K(+) groups remarkably increased compared with anti-miR-429-NC+p70S6K(+)-NC groups and the expressions of p70S6K, p-p70S6K, and p-S6K in anti-miR-429+p70S6K(-) groups increased compared with anti-miR-429-NC+p70S6K(-)-NC groups. Meanwhile the expressions of S6K had no obvious changes (**Figure 7D**). The above results proved that cotransfection of miR-429 and p70S6K reversely regulated p70S6K-S6 signaling pathway.

## Discussion

Although brain tumors can partially and unevenly destroy the BBB, and destroy its function, there is only a weak increase in vascular permeability in brain tumors (Siegal and Zylber-Katz, 2002). The reason is that although the tumor vessels have different structural and functional characteristics compared to the normal blood vessels, both exist BTB, which stops the delivery of cancer drugs into the cancer tissues through BTB (Erdlenbruch et al., 2003). Therefore, it is the key factor to improve the transport of drugs and increase the drugs concentration at the treatment site. EMAP-II is a proinflammatory cytokine that plays a role in mediating inflammatory responses (Lozhko et al., 2013). A number of studies have shown that EMAP-II is a tumor suppressor, for example, in fibrosarcoma (Haridas et al., 2008), pituitary adenomas (Bottoni et al., 2007), pancreatic cancer (Awasthi et al., 2010, 2011, 2013), gliomas (Liu et al., 2015). To date, the main research on EMAP-II has focused on its inflammatory mediators and anti-tumor effects, and EMAP-II also plays an important role in angiogenesis. EMAP-II has been shown to have a vital role in vascular contribution to lung development as an anti-angiogenic protein (Lal and Schwarz, 2014). In this study, we focus on the effect and mechanism of EMAP-II regulating the permeability of BTB. In this study, we confirmed that EMAP-II significantly increased the permeability of BTB models and decreased the expression of TJ-associated proteins (ZO-1, occludin and claudin-5) in human GECs, in a time- and dose-dependent manner. EMAP-II (0.05 nM) can influence the opening of the BTB. Previous experiments had shown that low-doses of EMAP-II can open the BTB in mice, most markedly after 1 h (Li Z. et al., 2015). In our human BTB models, the effect of EMAP-II (0.05 nM) at 0.5, 1, and 2 h caused a significant increase in the BTB permeability, and expression of TJ-associated proteins (ZO-1, occludin and claudin-5) was down-regulated, most notably at 1 h. Most studies have reported that effects on barrier function mostly occur between 24 and 48 h. A curious phenomenon was found here, namely a very quick and rapidly reversible effect of EMAP-II on barrier function. Mullin and Snock (1990) reported a very similar time course in their *in vitro* models, demonstrating that the effect of TNF on transepithelial resistance occurred at 90 min after cell sheet exposure to TNF.

Human miR-429 is a miR-200 family member, located in 1p36.33, and is involved in the occurrence and development of tumors. miR-429 has been reported to promote tumor development in liver cancer (Huang et al., 2013). However, most studies have demonstrated that miR-429 behaves as a tumor suppressor, for example, in non-small cell lung cancer (Zhu et al., 2014), renal cell carcinoma (Chen et al., 2016), esophageal cancer (Wang et al., 2013) and many kinds of malignant cancers (Fan et al., 2016; Wang et al., 2016). In addition, miR-429 inhibited the invasion of gliomas by BMK1 suppression (Chen et al., 2015). In human ECs, miR-429 attenuated HIF-1 activities, and thus influenced HIF1A and VEGFA mRNA (Bartoszewska et al., 2015). A previous report showed that miR-200b influenced BTB permeability by targeting ROCKII and RhoA (Ma and Xue, 2016). As a member of the same family, miR-429 may influence BTB permeability.

This study showed that miR-429 expression in human GECs was significantly lower than in human microvascular ECs; furthermore, miR-429 was weakly expressed in gliomas and was negatively correlated with different pathological grades of gliomas. This suggested that miR-429 plays a role as a tumor suppressor gene in gliomas, and regulates vascular function; however, whether or not EMAP-II influences the BTB permeability is unclear. Our results showed that miR-429 may be involved in the process of EMAP-II-induced regulation of BTB permeability. EMAP-II increased miR-429 expression in GECs in *in vitro* BTB models; and miR-429 expression increased at 0.5, 1, and 2 h, of which the most significant time point was 1 h. EMAP-II increased miR-429 expression in GECs in *in vitro* BTB models. There was a time-dependent trend, and a consistent increase of BTB permeability and a decrease of TJ-associated protein expression (ZO-1, occludin and claudin-5). This showed that miR-429 could participate in the mechanism of EMAP-II in *in vitro* BTB models.

To verify whether miR-429, as a tumor-inhibiting factor, could regulate BTB permeability, we successfully established stably transfected cell lines of miR-429 overexpression and silencing, and we then built *in vitro* BTB models. Firstly, TEER values and HRP fluxes were tested to evaluate the permeability of the BTB. Results showed that miR-429 overexpression significantly increased BTB permeability, and miR-429 silencing significantly decreased BTB permeability. In addition, it was found that some miRNA (miR-181a, miR-148b-3p, and miR-140), as tumor-inhibiting factors in glioma, were consistent with our results in the regulation of BTB permeability (Ma et al., 2014, 2016; Sa et al., 2017). This seems to be an oxymoron for a “tumor suppressor gene.” Why does it happen? The mechanism is not clear, and we need to further study.

TJ-associated proteins (ZO-1, occludin and claudin-5) are important parts of TJ, and their decreased expressions could significantly open TJ and increase BTB permeability. In this study, the results of qRT-PCR and western blotting showed miR-429 overexpression greatly down-regulated mRNA and protein expression of ZO-1, occludin and claudin-5; conversely, miR-429 silencing induced up-regulation of ZO-1, occludin and claudin-5. The results of immunofluorescence and western blotting were consistent. These results indicated that miR-429 increased the BTB permeability by down-regulating TJ-associated protein expression.

It is unclear how miR-429 down-regulates the expression of TJ-associated proteins. In this study, bioinformatics software Human Release TargetScan 6.2 was applied to predict the potential targets of miR-429. The results showed that there was one potential binding site at 71–77 of the ZO-1 mRNA 3′UTR and there were two potential binding sites at 139–145 and 2151–2157 of the occludin mRNA 3′UTR. The sequences of the above mentioned sites and the miR-429 seed region were shown to be complementary. The dual luciferase reporter gene assays confirmed that miR-429 could target TJ-associated proteins (ZO-1 and occludin), and their binding sites were verified. The results confirmed that ZO-1 and occludin were the target genes of miR-429. Bioinformatics software was also used to predict potential binding sites at the 3′UTR of claudin-5 mRNA and miR-429, but the results were negative. Nevertheless, the results of western blotting showed that miR-429 overexpression decreased the expression of claudin-5, and miR-429 silencing increased the expression of claudin-5, which indicated that miR-429 regulated the expression of claudin-5 by different mechanisms, which still needs further research.

TNF is one of the strongest bioactive agents that has been discovered to kill tumors directly; moreover, there is evidence that TNF can decrease TJs and barrier integrity (Mullin and Snock, 1990; Takigawa et al., 2017). Similar to TNF, EMAP-II, as an anti-angiogenesis cytokine, can inhibit the growth of various tumors, and can cause a decrease in TJs. Although studies have shown that the opening of TJs and the increase of BTB permeability can lead to the tumor growth, this was shown to be a slow process, requiring at least several weeks (Mullin et al., 1996; Soler et al., 1999). However, TNF and EMAP-II could induce a decrease in TJs and thus increase BTB permeability in 1 or 2 h, with a rapid recovery. We showed that EMAP-II could indeed reversibly increase the permeability of BTB in a short time (1 or 2 h), which helped delivery of anti-tumor drugs into the brain tissues, while at the same time inhibiting tumor growth. There are differences in the structure and function between the BBB and the BTB. In this study, we focused on the BTB. As tumor tissues cause partial destruction of the BTB, its permeability tends to be greater than that of the BBB. However, the presence of the BTB tends to restrict the entry of chemotherapeutic drugs into tumor tissues. miR-429 is a tumor suppressor gene, and its overexpression decreased expression of TJ-associated proteins and increased the BTB permeability. For example, a previous study by our team found that miR-181a was a tumor suppressor gene that also opened the BTB (Ma et al., 2014).

P70S6K functions as part of a signaling pathway including mTOR, which is the target of rapamycin. P70S6K plays a biological role by phosphorylating S6 protein kinase to promote translation of mRNA with 5′TOP (In et al., 2016). P70S6K is involved in tumor proliferation, apoptosis and angiogenesis (Ai et al., 2015; Lamour et al., 2015; Liu et al., 2016). P70S6K plays an oncogenic role in the malignant behavior of tumors, for example, in non-small cell lung cancer (In et al., 2016; Qiu et al., 2016), breast cancer (Esteva et al., 2010), pancreatic cancer (Chai et al., 2015), acute lymphoblastic leukemia (Li H. et al., 2015), ovarian cancer (Ataie-Kachoie et al., 2015) and glioma (Zhang et al., 2015). Secalonic acid-D (Guru et al., 2015), Tanshinone IIA (Li G. et al., 2015), celastrol (Han et al., 2014), α-santalol (Saraswati et al., 2013) and Siegesbeckia (Xiao et al., 2016) exhibited potent antiangiogenic activities by regulating p70S6K. However, whether p70S6K is involved in regulating EMAP-II-induced opening of the BTB in gliomas has not been reported.

Results from qRT-PCR and western blotting showed that EMAP-II can down-regulate p70S6K expression, which was consistent with TJ-associated proteins, suggesting that EMAP-II can affect TJ-associated proteins by regulating p70S6K. To determine whether EMAP-II can regulate p70S6K, by means of miR-429, we established miR-429 overexpression and silencing BTB models, followed by detection of p70S6K expression in GECs; the results showed that miR-429 overexpression significantly reduced p70S6K protein expression, while down-regulating its phosphorylation level. Conversely, miR-429 silencing significantly up-regulated p70S6K expression, as well as increasing phosphorylation of p70S6K. Bioinformatics software TargetScan Human Release 6.2 was used to predict that there was a potential binding site at 291–298 of p70S6K mRNA3′UTR. The dual luciferase reporter gene system analysis confirmed that miR-429 directly targeted p70S6K, and the binding site was confirmed. The above results showed that p70S6K was a target gene of miR-429. However, it was unclear whether p70S6K is involved in miR-429 regulation of TJ-associated protein expression.

To elucidate the effect of p70S6K on the expression of TJ-associated proteins, we successfully established stably transfected GECs with p70S6K overexpression and silencing. TEER values and HRP flux were used to demonstrate that p70S6K overexpression significantly decreased the permeability of the BTB, and p70S6K silencing significantly increased the permeability of the BTB models, which confirmed that p70S6K influenced the BTB permeability. Western blotting was used to show that when p70S6K was overexpressed in GECs, the expression levels of TJ-associated proteins (ZO-1, occludin and claudin-5) increased; p70S6K silencing, on the other hand, decreased the same proteins expression, which demonstrated that p70S6K affected permeability by influencing TJ-associated proteins.

BTB models with cotransfection of miR-429 and p70S6K were constructed *in vitro*. TEER values and HRP flux were used to test changes in BTB permeability, and western blotting was carried out to test the changes of expression levels of ZO-1, occludin and claudin-5 in GECs of the BTB. The results confirmed that miR-429 affected the expression of ZO-1, occludin and claudin-5 by targeting p70S6K, thus affecting BTB permeability. Overexpression of miR-429 greatly down-regulated p70S6K expression, as well as the phosphorylation levels, while miR-429 silencing significantly up-regulated p70S6K expression, and increased phosphorylation levels. miR-429 knockdown increased the expression of ZO-1, occludin and claudin-5. Therefore, miR-429 targeted p70S6K, down-regulated p70S6K expression and phosphorylation levels, and further affected phosphorylation levels of S6, which consequently increased BTB permeability.

In summary, this study demonstrated that miR-429 is an important regulator of EMAP-II, and influenced BTB permeability. EMAP-II can significantly up-regulate miR-429 expression; subsequently the up-regulated miR-429 inhibited the expression of TJ-associated proteins, by two mechanisms, in order to influence BTB permeability. Firstly, up-regulated miR-429 inhibited the expression of TJ-associated proteins by negative targeting, resulting in an increase in BTB permeability. Secondly, up-regulated miR-429 inhibited the expression and phosphorylation levels of p70S6K by negative targeting, and also decreased S6 phosphorylation, causing down-regulation of TJ-associated proteins and thus an increase in BTB permeability. These results demonstrate that EMAP-II and miR-429 are potential tools for increasing BTB permeability, which could be exploited therapeutically.

## Author Contributions

YL, LL, and YX designed the research. LC performed the majority of the experiments. LC, JZ, XL, JL, JC, ZL, ZX, HT, and PW contributed to collection and assembly of data. LC, and LL wrote the paper. YL, LL, and YX supervised the research.

## Conflict of Interest Statement

The authors declare that the research was conducted in the absence of any commercial or financial relationships that could be construed as a potential conflict of interest.

## Footnotes

1 www.targetscan.org

2 www.targetscan.org

3 www.targetscan.org

## Abbreviations

BBB: blood–brain barrier
BTB: blood-tumor barrier
ECs: endothelial cells
EMAP-II: endothelial monocyte activating polypeptide-II
EMT: epithelial mesenchymal transition
GECs: glioma vascular endothelial cells
HGG: high-grade glioma
HRP: horseradish peroxidase
LGG: low-grade glioma
miR-429: microRNA-429
NBT: normal brain tissue
TEER: transepithelial electric resistance
TJ: tight junction.

## References

[B1] Ai F. Chen M. Yu B. Yang Y. Xu G. Gui F. (2015). Berberine regulates proliferation, collagen synthesis and cytokine secretion of cardiac fibroblasts via AMPK-mTOR-p70S6K signaling pathway. *Int. J. Clin. Exp. Pathol.* 8 12509–12516. 26722438 PMC4680383

[B2] Ataie-Kachoie P. Pourgholami M. H. Bahrami B. F. Badar S. Morris D. L. (2015). Minocycline attenuates hypoxia-inducible factor-1alpha expression correlated with modulation of p53 and AKT/mTOR/p70S6K/4E-BP1 pathway in ovarian cancer: in vitro and in vivo studies. *Am. J. Cancer Res.* 5 575–588. 25973298 PMC4396050

[B3] Awasthi N. Schwarz M. A. Schwarz R. E. (2010). Combination effects of bortezomib with gemcitabine and EMAP II in experimental pancreatic cancer. *Cancer Biol. Ther.* 10 99–107. 10.4161/cbt.10.1.12169 20495354

[B4] Awasthi N. Schwarz M. A. Schwarz R. E. (2011). Enhancing cytotoxic agent activity in experimental pancreatic cancer through EMAP II combination therapy. *Cancer Chemother. Pharmacol.* 68 571–582. 10.1007/s00280-010-1514-7 21110024

[B5] Awasthi N. Zhang C. Hinz S. Schwarz M. A. Schwarz R. E. (2013). Enhancing sorafenib-mediated sensitization to gemcitabine in experimental pancreatic cancer through EMAP II. *J. Exp. Clin. Cancer Res.* 32:12. 10.1186/1756-9966-32-12 23497499 PMC3618297

[B6] Bartoszewska S. Kochan K. Piotrowski A. Kamysz W. Ochocka R. J. Collawn J. F. (2015). The hypoxia-inducible miR-429 regulates hypoxia-inducible factor-1alpha expression in human endothelial cells through a negative feedback loop. *FASEB J.* 29 1467–1479. 10.1096/fj.14-267054 25550463 PMC4396612

[B7] Black K. L. Ningaraj N. S. (2004). Modulation of brain tumor capillaries for enhanced drug delivery selectively to brain tumor. *Cancer Control* 11 165–173. 10.1177/107327480401100304 15153840

[B8] Bottoni A. Vignali C. Piccin D. Tagliati F. Luchin A. Zatelli M. C. (2007). Proteasomes and RARS modulate AIMP1/EMAP II secretion in human cancer cell lines. *J. Cell. Physiol.* 212 293–297. 10.1002/jcp.21083 17443684

[B9] Cai H. Liu W. Xue Y. Shang X. Liu J. Li Z. (2015a). Roundabout 4 regulates blood-tumor barrier permeability through the modulation of ZO-1, Occludin, and Claudin-5 expression. *J. Neuropathol. Exp. Neurol.* 74 25–37. 10.1097/NEN.0000000000000146 25470344

[B10] Cai H. Xue Y. Wang P. Wang Z. Li Z. Hu Y. (2015b). The long noncoding RNA TUG1 regulates blood-tumor barrier permeability by targeting miR-144. *Oncotarget* 6 19759–19779. 10.18632/oncotarget.4331 26078353 PMC4637319

[B11] Chai X. Chu H. Yang X. Meng Y. Shi P. Gou S. (2015). Metformin increases sensitivity of pancreatic cancer cells to gemcitabine by reducing CD133^+^ cell populations and suppressing ERK/P70S6K signaling. *Sci. Rep.* 5:14404. 10.1038/srep14404 26391180 PMC4585731

[B12] Chen D. Li Y. Li Y. Jin L. Su Z. Yu Z. (2016). Tumor suppressive microRNA429 regulates cellular function by targeting VEGF in clear cell renal cell carcinoma. *Mol. Med. Rep.* 13 1361–1366. 10.3892/mmr.2015.4653 26647818

[B13] Chen W. Zhang B. Guo W. Gao L. Shi L. Li H. (2015). miR-429 inhibits glioma invasion through BMK1 suppression. *J. Neurooncol.* 125 43–54. 10.1007/s11060-015-1887-x 26272601

[B14] Dong S. J. Cai X. J. Li S. J. (2016). The clinical significance of MiR-429 as a predictive biomarker in colorectal cancer patients receiving 5-fluorouracil treatment. *Med. Sci. Monit.* 22 3352–3361. 10.12659/MSM.900674 27654003 PMC5036382

[B15] Erdlenbruch B. Schinkhof C. Kugler W. Heinemann D. E. Herms J. Eibl H. (2003). Intracarotid administration of short-chain alkylglycerols for increased delivery of methotrexate to the rat brain. *Br. J. Pharmacol.* 139 685–694. 10.1038/sj.bjp.0705302 12812991 PMC1573898

[B16] Esteva F. J. Guo H. Zhang S. Santa-Maria C. Stone S. Lanchbury J. S. (2010). PTEN, PIK3CA, p-AKT, and p-p70S6K status: association with trastuzumab response and survival in patients with HER2-positive metastatic breast cancer. *Am. J. Pathol.* 177 1647–1656. 10.2353/ajpath.2010.090885 20813970 PMC2947262

[B17] Fan J. Y. Fan Y. J. Wang X. L. Xie H. Gao H. J. Zhang Y. (2016). miR-429 is involved in regulation of NF-kappaBactivity by targeting IKKbeta and suppresses oncogenic activity in cervical cancer cells. *FEBS Lett.* 591 118–128. 10.1002/1873-3468.12502 27883176

[B18] Guru S. K. Pathania A. S. Kumar S. Ramesh D. Kumar M. Rana S. (2015). Secalonic Acid-D represses HIF1alpha/VEGF-mediated angiogenesis by regulating the Akt/mTOR/p70S6K signaling cascade. *Cancer Res.* 75 2886–2896. 10.1158/0008-5472.CAN-14-2312 25977334

[B19] Han X. Sun S. Zhao M. Cheng X. Chen G. Lin S. (2014). Celastrol stimulates hypoxia-inducible factor-1 activity in tumor cells by initiating the ROS/Akt/p70S6K signaling pathway and enhancing hypoxia-inducible factor-1alpha protein synthesis. *PLOS ONE* 9:e112470. 10.1371/journal.pone.0112470 25383959 PMC4226555

[B20] Haridas S. Bowers M. Tusano J. Mehojah J. Kirkpatrick M. Burnham D. K. (2008). The impact of Meth A fibrosarcoma derived EMAP II on dendritic cell migration. *Cytokine* 44 304–309. 10.1016/j.cyto.2008.09.002 18951814

[B21] Huang X. Y. Yao J. G. Huang H. D. Wang C. Ma Y. Xia Q. (2013). MicroRNA-429 modulates hepatocellular carcinoma prognosis and tumorigenesis. *Gastroenterol. Res. Pract.* 2013:804128. 10.1155/2013/804128 24204382 PMC3800573

[B22] In J. K. Kim J. K. Oh J. S. Seo D. W. (2016). 5-Caffeoylquinic acid inhibits invasion of non-small cell lung cancer cells through the inactivation of p70S6K and Akt activity: involvement of p53 in differential regulation of signaling pathways. *Int. J. Oncol.* 48 1907–1912. 10.3892/ijo.2016.3436 26984670

[B23] Lal C. V. Schwarz M. A. (2014). Vascular mediators in chronic lung disease of infancy: role of endothelial monocyte activating polypeptide II (EMAP II). *Birth Defects Res. A Clin. Mol. Teratol.* 100 180–188. 10.1002/bdra.23234 24619875 PMC4280095

[B24] Lamour V. Henry A. Kroonen J. Nokin M. J. von Marschall Z. Fisher L. W. (2015). Targeting osteopontin suppresses glioblastoma stem-like cell character and tumorigenicity in vivo. *Int. J. Cancer* 137 1047–1057. 10.1002/ijc.29454 25620078

[B25] Li G. Shan C. Liu L. Zhou T. Zhou J. Hu X. (2015). Tanshinone IIA inhibits HIF-1alpha and VEGF expression in breast cancer cells via mTOR/p70S6K/RPS6/4E-BP1 signaling pathway. *PLOS ONE* 10:e0117440. 10.1371/journal.pone.0117440 25659153 PMC4320086

[B26] Li H. Kong X. Cui G. Ren C. Fan S. Sun L. (2015). Rapamycin restores p14, p15 and p57 expression and inhibits the mTOR/p70S6K pathway in acute lymphoblastic leukemia cells. *Int. J. Hematol.* 102 558–568. 10.1007/s12185-015-1858-1 26362858

[B27] Li Z. Liu X. B. Liu Y. H. Xue Y. X. Liu J. Teng H. (2016a). Low-dose endothelial monocyte-activating polypeptide-II increases blood-tumor barrier permeability by activating the RhoA/ROCK/PI3K signaling pathway. *J. Mol. Neurosci.* 59 193–202. 10.1007/s12031-015-0668-5 26521255

[B28] Li Z. Liu X. B. Liu Y. H. Xue Y. X. Liu J. Teng H. (2016b). Low-dose endothelial monocyte-activating polypeptide-II induces blood-tumor barrier opening via the cAMP/PKA/Rac1 pathway. *J. Mol. Neurosci.* 58 153–161. 10.1007/s12031-015-0649-8 26358039

[B29] Li Z. Liu X. B. Liu Y. H. Xue Y. X. Wang P. Liu L. B. (2015). Roles of serine/threonine phosphatases in low-dose endothelial monocyte-activating polypeptide-II-induced opening of blood-tumor barrier. *J. Mol. Neurosci.* 57 11–20. 10.1007/s12031-015-0604-8 26087743

[B30] Liu G. Zhong M. Guo C. Komatsu M. Xu J. Wang Y. (2016). Autophagy is involved in regulating influenza A virus RNA and protein synthesis associated with both modulation of Hsp90 induction and mTOR/p70S6K signaling pathway. *Int. J. Biochem. Cell Biol.* 72 100–108. 10.1016/j.biocel.2016.01.012 26794463

[B31] Liu L. B. Xie H. Xue Y. X. Liu Y. H. Li Z. Wang P. (2015). Endothelial-monocyte-activating polypeptide II induces rat C6 glioma cell apoptosis via the mitochondrial pathway. *Biochem. Biophys. Res. Commun.* 457 595–601. 10.1016/j.bbrc.2015.01.030 25600803

[B32] Lozhko D. Stanek J. Kazimierczuk K. Zawadzka-Kazimierczuk A. Kozminski W. Zhukov I. (2013). (1)H, (13)C, and (15)N chemical shifts assignments for human endothelial monocyte-activating polypeptide EMAP II. *Biomol. NMR Assign.* 7 25–29. 10.1007/s12104-012-9369-y 22392337

[B33] Ma J. Wang P. Yao Y. Liu Y. Li Z. Liu X. (2016). Knockdown of long non-coding RNA MALAT1 increases the blood-tumor barrier permeability by up-regulating miR-140. *Biochim. Biophys. Acta* 1859 324–338. 10.1016/j.bbagrm.2015.11.008 26619802

[B34] Ma J. Yao Y. Wang P. Liu Y. Zhao L. Li Z. (2014). MiR-181a regulates blood-tumor barrier permeability by targeting Kruppel-like factor 6. *J. Cereb. Blood Flow Metab.* 34 1826–1836. 10.1038/jcbfm.2014.152 25182666 PMC4269760

[B35] Ma T. Xue Y. X. (2016). MiRNA-200b regulates RMP7-induced increases in blood-tumor barrier permeability by targeting RhoA and ROCKII. *Front. Mol. Neurosci.* 9:9. 10.3389/fnmol.2016.00009 26903801 PMC4742559

[B36] Miyoshi J. Takai Y. (2005). Molecular perspective on tight-junction assembly and epithelial polarity. *Adv. Drug Deliv. Rev.* 57 815–855. 10.1016/j.addr.2005.01.008 15820555

[B37] Mogylnytska L. A. (2015). Endothelial monocyte-activating polypeptide-II: properties, functions, and pathogenetic significance. *Fiziol. Zh.* 61 102–111. 10.15407/fz61.01.102 26040042

[B38] Mullin J. M. Snock K. V. (1990). Effect of tumor necrosis factor on epithelial tight junctions and transepithelial permeability. *Cancer Res.* 50 2172–2176. 2180562

[B39] Mullin J. M. Soler A. P. Laughlin K. V. Kampherstein J. A. Russo L. M. Saladik D. T. (1996). Chronic exposure of LLC-PK1 epithelia to the phorbol ester TPA produces polyp-like foci with leaky tight junctions and altered protein kinase C-alpha expression and localization. *Exp. Cell Res.* 227 12–22. 10.1006/excr.1996.0244 8806446

[B40] Nakagawa S. Deli M. A. Nakao S. Honda M. Hayashi K. Nakaoke R. (2007). Pericytes from brain microvessels strengthen the barrier integrity in primary cultures of rat brain endothelial cells. *Cell Mol. Neurobiol.* 27 687–694. 10.1007/s10571-007-9195-4 17823866 PMC11517186

[B41] Park J. Aryal M. Vykhodtseva N. Zhang Y. Z. McDannold N. (2016). Evaluation of permeability, doxorubicin delivery, and drug retention in a rat brain tumor model after ultrasound-induced blood-tumor barrier disruption. *J. Control Release* 250 77–85. 10.1016/j.jconrel.2016.10.011 27742444 PMC5384106

[B42] Park S. M. Gaur A. B. Lengyel E. Peter M. E. (2008). The miR-200 family determines the epithelial phenotype of cancer cells by targeting the E-cadherin repressors ZEB1 and ZEB2. *Genes Dev.* 22 894–907. 10.1101/gad.1640608 18381893 PMC2279201

[B43] Pecot C. V. Rupaimoole R. Yang D. Akbani R. Ivan C. Lu C. (2013). Tumour angiogenesis regulation by the miR-200 family. *Nat. Commun.* 4:2427. 10.1038/ncomms3427 24018975 PMC3904438

[B44] Qiu Z. X. Sun R. F. Mo X. M. Li W. M. (2016). The p70S6K specific inhibitor PF-4708671 impedes non-small cell lung cancer growth. *PLOS ONE* 11:e0147185. 10.1371/journal.pone.0147185 26771549 PMC4714881

[B45] Sa L. Li Y. Zhao L. Liu Y. Wang P. Liu L. (2017). The role of HOTAIR/miR-148b-3p/USF1 on regulating the permeability of BTB. *Front. Mol. Neurosci.* 10:194. 10.3389/fnmol.2017.00194 28701916 PMC5487514

[B46] Salvador E. Burek M. Forster C. Y. (2016). Tight junctions and the tumor microenvironment. *Curr. Pathobiol. Rep.* 4 135–145. 10.1007/s40139-016-0106-6 27547510 PMC4978755

[B47] Saraswati S. Kumar S. Alhaider A. A. (2013). alpha-santalol inhibits the angiogenesis and growth of human prostate tumor growth by targeting vascular endothelial growth factor receptor 2-mediated AKT/mTOR/P70S6K signaling pathway. *Mol. Cancer.* 12:147. 10.1186/1476-4598-12-147 24261856 PMC4221991

[B48] Schwarz R. E. Schwarz M. A. (2004). In vivo therapy of local tumor progression by targeting vascular endothelium with EMAP-II. *J. Surg. Res.* 120 64–72. 10.1016/j.jss.2003.10.005 15172191

[B49] Siegal T. Zylber-Katz E. (2002). Strategies for increasing drug delivery to the brain: focus on brain lymphoma. *Clin. Pharmacokinet.* 41 171–186. 10.2165/00003088-200241030-00002 11929318

[B50] Soler A. P. Miller R. D. Laughlin K. V. Carp N. Z. Klurfeld D. M. Mullin J. M. (1999). Increased tight junctional permeability is associated with the development of colon cancer. *Carcinogenesis* 20 1425–1431. 10.1093/carcin/20.8.1425 10426787

[B51] Stamatovic S. M. Johnson A. M. Keep R. F. Andjelkovic A. V. (2016). Junctional proteins of the blood-brain barrier: new insights into function and dysfunction. *Tissue Barriers* 4:e1154641. 10.1080/21688370.2016.1154641 27141427 PMC4836471

[B52] Takigawa M. Iida M. Nagase S. Suzuki H. Watari A. Tada M. (2017). Creation of a claudin-2 binder and its tight junction-modulating activity in a human intestinal model. *J. Pharmacol. Exp. Ther.* 363 444–451. 10.1124/jpet.117.242214 28928120

[B53] Wang F. Jiang C. Sun Q. Yan F. Wang L. Fu Z. (2016). Downregulation of miR429 and inhibition of cell migration and invasion in nasopharyngeal carcinoma. *Mol. Med. Rep.* 13 3236–3242. 10.3892/mmr.2016.4940 26936585

[B54] Wang Y. Li M. Zang W. Ma Y. Wang N. Li P. (2013). MiR-429 up-regulation induces apoptosis and suppresses invasion by targeting Bcl-2 and SP-1 in esophageal carcinoma. *Cell. Oncol.* 36 385–394. 10.1007/s13402-013-0144-6 23999873 PMC13012206

[B55] Weksler B. B. Subileau E. A. Perriere N. Charneau P. Holloway K. Leveque M. (2005). Blood-brain barrier-specific properties of a human adult brain endothelial cell line. *FASEB J.* 19 1872–1874. 10.1096/fj.04-3458fje 16141364

[B56] Xiao P. Liu W. Zhou H. (2016). miR-429 promotes the proliferation of non-small cell lung cancer cells via targeting DLC-1. *Oncol. Lett.* 12 2163–2168. 10.3892/ol.2016.4904 27602157 PMC4998573

[B57] Yoshino H. Enokida H. Itesako T. Tatarano S. Kinoshita T. Fuse M. (2013). Epithelial-mesenchymal transition-related microRNA-200s regulate molecular targets and pathways in renal cell carcinoma. *J. Hum. Genet.* 58 508–516. 10.1038/jhg.2013.31 23635949

[B58] Zanchi N. E. Lancha A. H. Jr. (2008). Mechanical stimuli of skeletal muscle: implications on mTOR/p70s6k and protein synthesis. *Eur. J. Appl. Physiol.* 102 253–263. 10.1007/s00421-007-0588-3 17940791

[B59] Zhang C. Yuan X. R. Li H. Y. Zhao Z. J. Liao Y. W. Wang X. Y. (2015). Anti-cancer effect of metabotropic glutamate receptor 1 inhibition in human glioma U87 cells: involvement of PI3K/Akt/mTOR pathway. *Cell. Physiol. Biochem.* 35 419–432. 10.1159/000369707 25613036

[B60] Zhang L. Wang H. Xu J. Zhu J. Ding K. (2014a). Inhibition of cathepsin S induces autophagy and apoptosis in human glioblastoma cell lines through ROS-mediated PI3K/AKT/mTOR/p70S6K and JNK signaling pathways. *Toxicol. Lett.* 228 248–259. 10.1016/j.toxlet.2014.05.015 24875536

[B61] Zhang L. Wang H. Zhu J. Ding K. Xu J. (2014b). FTY720 reduces migration and invasion of human glioblastoma cell lines via inhibiting the PI3K/AKT/mTOR/p70S6K signaling pathway. *Tumour Biol.* 35 10707–10714. 10.1007/s13277-014-2386-y 25070489

[B62] Zhao P. Li Q. Shi Z. Li C. Wang L. Liu X. (2015). GSK-3beta regulates tumor growth and angiogenesis in human glioma cells. *Oncotarget* 6 31901–31915. 10.18632/oncotarget.5043 26388612 PMC4741649

[B63] Zhao X. F. Gartenhaus R. B. (2009). Phospho-p70S6K and cdc2/cdk1 as therapeutic targets for diffuse large B-cell lymphoma. *Expert Opin. Ther. Targets* 13 1085–1093. 10.1517/14728220903103833 19614561

[B64] Zhu W. He J. Chen D. Zhang B. Xu L. Ma H. (2014). Expression of miR-29c, miR-93, and miR-429 as potential biomarkers for detection of early stage non-small lung cancer. *PLOS ONE* 9:e87780. 10.1371/journal.pone.0087780 24523873 PMC3921142

